# Associations between Antenatal Care Visit Attendance and Infant Mortality and Growth

**DOI:** 10.4269/ajtmh.23-0659

**Published:** 2024-04-16

**Authors:** Aimee J. Lansdale, Mamadou Bountogo, Ali Sie, Alphonse Zakane, Guillaume Compaoré, Thierry Ouedraogo, Elodie Lebas, Thomas Lietman, Catherine E. Oldenburg

**Affiliations:** ^1^ Department of Epidemiology and Biostatistics, University of California, San Francisco, San Francisco, California; ^2^ Centre de Recherche en Santé de Nouna, Nouna, Burkina Faso; ^3^ Francis I Proctor Foundation, University of California, San Francisco, San Francisco, California; ^4^ Department of Ophthalmology, University of California, San Francisco, San Francisco, California

## Abstract

This study examines the association between antenatal care (ANC) attendance and infant mortality and growth outcomes. The study used data from the Nouveux-nés et Azithromycine: une Innovation dans le Traitement des Enfants (NAITRE) trial conducted in Burkina Faso. This analysis included 21,795 neonates aged 8 to 27 days who were enrolled in the trial and had ANC data available. Infants were followed until 6 months of age. The analysis adjusted for potential confounders including infant’s sex, maternal age, education, urbanicity, geographic region, season (dry versus rainy), pregnancy type (singleton versus multiple), number of previous pregnancies, if the infant was breastfed, and if the facility had an onsite physician to account for level of care. We used logistic and linear regression models to evaluate the association between ANC visits and all-cause infant mortality and infant growth measurements at 6 months. There was no significant association between ANC visits and 6-month mortality. Higher ANC attendance was associated with improved growth outcomes in infants at 6 months of age. After adjusting for potential confounders, each additional ANC visit was associated with a 0.03 kg increase in mean weight, 0.07 cm increase in mean length, 0.04 SD increase in mean mid-upper-arm circumference, 0.04 SD increase in mean height-for-age, 0.04 SD mean weight-for-age, and 0.02 SD mean weight-for-length Z-scores. These mean differences were statistically significant (except for weight-for-length Z-scores) but may not be clinically meaningful. Further research is warranted to explore the relationship between ANC attendance and longer-term health outcomes among infants.

## INTRODUCTION

Increased antenatal care (ANC) attendance is associated with improved birth outcomes.^1^ In 2016, the WHO updated their recommendation of ANC contacts to reduce perinatal mortality and improve women’s experience of care from a minimum of four to eight visits.^2^ However, there is limited data on the of the uptake of the WHO’s recommendation in many countries, especially in low-resource settings. Although increased ANC attendance has been shown to be associated with improved birth outcomes, there is limited evidence of the longer-term association of ANC attendance on infant growth and mortality during the first year after birth.^3^^–^^5^

Antenatal care visits provide opportunities for mothers to receive health screenings, nutritional advice and supplements (e.g., folic acid), and preventative malaria treatment. In areas with high malaria transmission, ANC is especially important for mothers to receive preventative malaria treatment because malaria infections during pregnancy are associated with higher infant mortality due to their contribution to low birthweight (LBW) and premature delivery.^6^

In a previous analysis, mothers in Burkina Faso from the Nouveux-nés et Azithromycine: une Innovation dans le Traitement des Enfants (NAITRE) study who attended at least four ANC visits were less likely to give birth to a baby with LBW (defined by WHO as infants weighing <2,500 g at birth) compared with women attending fewer than four ANC visits.^5^^,^^7^ Low birthweight contributes to many poor health outcomes, including fetal and neonatal mortality.^8^ If they survive, LBW newborns are also more likely to develop noncommunicable diseases later in life such as obesity and diabetes.^9^^,^^10^ In addition, children <10 years of age who were LBW have been found to have lower cognitive and motor development scores compared with children with normal weight.^11^

Using data from the NAITRE trial, this study aimed to examine whether ANC attendance is associated with infant mortality and growth outcomes up to 6 months of age.^12^^,^^13^ We hypothesized that higher ANC attendance will be associated with lower mortality and better growth outcomes during the first 6 months of life among infants in the NAITRE trial in Burkina Faso. Given that the bulk of the evidence relating to ANC focuses on birth outcomes, this analysis fills a gap in the literature by evaluating longer-term outcomes of greater ANC attendance.

## MATERIALS AND METHODS

The NAITRE trial was a 1:1 double-masked randomized placebo-controlled trial in five regions of Burkina Faso that evaluated the efficacy of a single oral 20 mg/kg dose of azithromycin compared with a matching placebo in reducing all-cause infant mortality at 6 months of age for 21,832 neonates aged 8 to 27 days.^12^^,^^13^ The study was conducted in 44 Centers de Santé et de Promotion Sociale and Centers Médicaux, primary healthcare facilities that are the first level of healthcare in the country, in the following five regions of Burkina Faso: Center, Center Ouest, Boucle du Mouhoun, Hauts-Bassins, and Cascade.^12^^,^^13^ To be eligible to participate in the study, neonates had to weigh at least 2,500 g at the time of enrollment (8–27 days of age), be able to feed orally, and not have clinical signs of jaundice.^11^^,^^12^ They were then randomized to a single, directly observed, oral dose of azithromycin or matching placebo.^12^^,^^13^ Although LBW newborns were not excluded from the study, the infant had to reach 2,500 g by the time they were 27 days old to participate in the NAITRE trial and this analysis. The research was reviewed and received approval from both the Comité d’Ethique pour la Recherche en Santé in Ouagadougou, Burkina Faso (Protocol No. 2018-10-123) and the Institutional Review Board at the University of California, San Francisco (Protocol No. 18-25027). Before participation, written informed consent was obtained from at least one parent or guardian of each enrolled infant. Infants were enrolled from April 2019 to December 2020, and the last follow-up visit occurred in July 2021.

Using a standardized questionnaire, trained study nurses in Burkina Faso collected health data on the mothers and infants. At baseline, the data collected included infant’s sex, maternal age in years, mother’s education level, region, season of enrollment (dry versus rainy), pregnancy type, number of children in the household, and whether the infant was breastfed (collected at enrollment, ages 8–27 days). The study nurses also assessed vital status and collected anthropometric measurements at baseline and 6 months of age. Weight in kilograms was measured using a standardized digital scale. Length in centimeters was measured using a Shorrboard (Weigh and Measure, LLC, Olney, MD), and the median of three measurements was used for analysis. Mid-upper-arm circumference (MUAC) was measured using a standard MUAC tape, and the median of three consecutive measurements was used for analysis. Data on the exposure, ANC attendance, was extracted from each infant’s government-issued health card (*carnet de santé*). Healthcare for pregnant women and children under 5 years of age is free in Burkina Faso. A health card for each child is issued during pregnancy, during which key information such as ANC attendance, birthweight, and other measures are recorded. During the enrollment visit for the study, the study nurse recorded the number of ANC visits in the study’s mobile data collection application using information from the health card. Infants were followed at 6 months of age in the clinic of enrollment. If an infant did not return for the follow-up visit, attempts were made to contact the family to arrange a home visit. For infants for whom a home visit was not possible, vital status was assessed via phone call. Anthropometric measurements were not available for infants who were followed via phone call.

Because categorizing variables can result in a loss of information, ANC attendance was analyzed as a continuous variable. The study outcomes included all-cause mortality at 6 months measured by vital status and growth outcomes at 6 months. The growth measurements at 6 months of age included weight, length, MUAC, length-for-age Z-score (LAZ), weight-for-age Z-score (WAZ), and weight-for-length Z-Score (WLZ) based on 2006 WHO growth standards. The LAZ (< –2 SD), WAZ (< –2 SD), and WLZ (< –2 SD) were used as criterions for stunting, underweight, and wasting, in this respective order.^14^ The Z-scores were calculated in R using the “growthstandards” package by the NAITRE data team. To determine the association of ANC visit attendance on mortality and growth, we used univariate and multivariable logistic regression analyses expressed as odds ratios (ORs) for the mortality outcome and linear regression models expressed as mean differences for the growth outcomes. An alpha level of <0.05 was considered statistically significant.

Using existing literature and a directed acyclic graph, potential confounders of the association between ANC visit attendance and mortality and growth outcomes were determined before the data analysis was conducted. The following covariates were adjusted for: infant’s sex, maternal age, maternal education, the urbanicity of the facility (urban, peri-urban, and rural), geographic region of the facility, season (dry versus rainy), pregnancy type (singleton versus multiple), the number of previous pregnancies, if the infant was breastfed, and if the facility had an onsite physician to account for level of care. Although we did not have socioeconomic information of the households, we tried to account for the impact socioeconomic status (SES) may have on ANC by accounting for the urbanicity of the facility. In this setting, “urban” was defined as living in a town with running water and electricity, “peri-urban” was defined as living in the outskirts of a town without running water or electricity, and “rural” was defined as living outside of a town without running water or electricity. We did not adjust for the NAITRE treatment arms of azithromycin or placebo because exposure to these groups occurred after ANC attendance. Adjusting for these treatment arms could lead to bias, and the treatment arm by definition cannot confound the relationship between ANC attendance and infant outcomes.

We conducted a sensitivity analysis in which we ran the same models among the placebo group only to understand the trends within the general population who were not receiving the active intervention. We also conducted a sensitivity analysis where ANC visit attendance was dichotomized into the following two categories: zero to three visits and four or more visits. These categories were determined by the previous WHO ANC recommendations.^2^ All analyses were conducted using Stata version 17.0 (StataCorp, College Station, TX). We also used R to create boxplots to visually examine the associations of ANC visit attendance on 6-month growth outcomes and calculate Z-scores.

## RESULTS

Of the 21,832 infants randomized into the NAITRE study, 21,727 had ANC data available from their government-issued health card (Table 1). Demographic characteristics of mothers and infants were similar among those with less than four ANC visits and those with four or more ANC visits (Table 1).

**Table 1:** Descriptive characteristics of study sample by antenatal care visit attendance (low antenatal care attendance (zero to three) versus high antenatal care attendance (four or more)

Characteristics	Total	Antenatal (zero to three)	Antenatal (four or more)
	*N* = 21,727	*n* = 6,869	*n* = 14,858
Infant’s sex			
Female	10,791 (50%)	3,470 (51%)	7,321 (49%)
Male	10,936 (50%)	3,399 (49%)	7,537 (51%)
Maternal age in years (mean, SD)	26 (6.17)	26 (6.27)	26 (6.13)
Maternal education level			
None	11,888 (55%)	4,474 (65%)	7,414 (50%)
Primary	3,940 (18%)	1,040 (15%)	2,900 (20%)
Secondary	5,289 (24%)	1,270 (18%)	4,019 (27%)
Secondary+	610 (3%)	85 (1%)	525 (4%)
Urbanicity			
Rural	3,799 (17%)	1,578 (23%)	2,221 (15%)
Urban	16,491 (76%)	4,694 (68%)	11,797 (79%)
Peri-urban	1,437 (7%)	597 (9%)	840 (6%)
Region			
Boucle du Mouhoun	2,573 (12%)	798 (12%)	1,775 (12%)
Cascade	3,978 (18%)	1,214 (18%)	2,764 (19%)
Center	1,865 (9%)	742 (11%)	1,123 (8%)
Center Ouest	2,427 (11%)	639 (9%)	1,788 (12%)
Hauts-Bassins	10,884 (50%)	3,476 (51%)	7,408 (50%)
Season			
Rainy	10,432 (48%)	3,383 (49%)	7,049 (47%)
Dry	11,295 (52%)	3,486 (51%)	7,809 (53%)
Pregnancy type			
Singleton	21,355 (98%)	6,747 (98%)	14,608 (98%)
Multiple	372 (2%)	122 (2%)	250 (2%)
Number of children in household			
0	6,382 (29%)	1,690 (25%)	4,692 (32%)
1	5,246 (24%)	1,642 (24%)	3,604 (24%)
2	4,181 (19%)	1,366 (20%)	2,815 (19%)
3 or more	5,918 (27%)	2,171 (32%)	3,747 (25%)
Breastfed			
No	26 (0%)	6 (0%)	20 (0%)
Yes	21,701 (100%)	6,863 (100%)	14,838 (100%)
Onsite physician			
No	19,046 (88%)	6,072 (88%)	12,974 (87%)
Yes	2,681 (12%)	797 (12%)	1,884 (13%)

Among enrolled infants, 50% were female and 50% were male. The mean maternal age in years was 26, and most mothers had no education (55%). The majority of the mothers and infants resided in an urban area (76%). Most infants enrolled in the trial were singleton (98%). The number of other children in the household was almost evenly split among the different categories (0, 1, 2, and 3 or more). Nearly all the infants in the study were breastfed at baseline (99.7%). Almost every mother in the study attended at least one ANC, with only 59 mothers not attending any visits (0.3%). Three women attended eight ANC visits.

Among infants with ANC data who were not lost to follow-up (*N* = 20,835), 92 died by the 6 months of age. In the adjusted model, we found no statistically significant evidence of an association between ANC attendance and infant mortality (adjusted OR 0.89, 95% CI 0.73–1.09). We found similar results in the sensitivity analysis restricting to the placebo group (adjusted OR 0.99, 95% CI 0.77–1.28; Supplemental Table 1).

**Figure 1.**
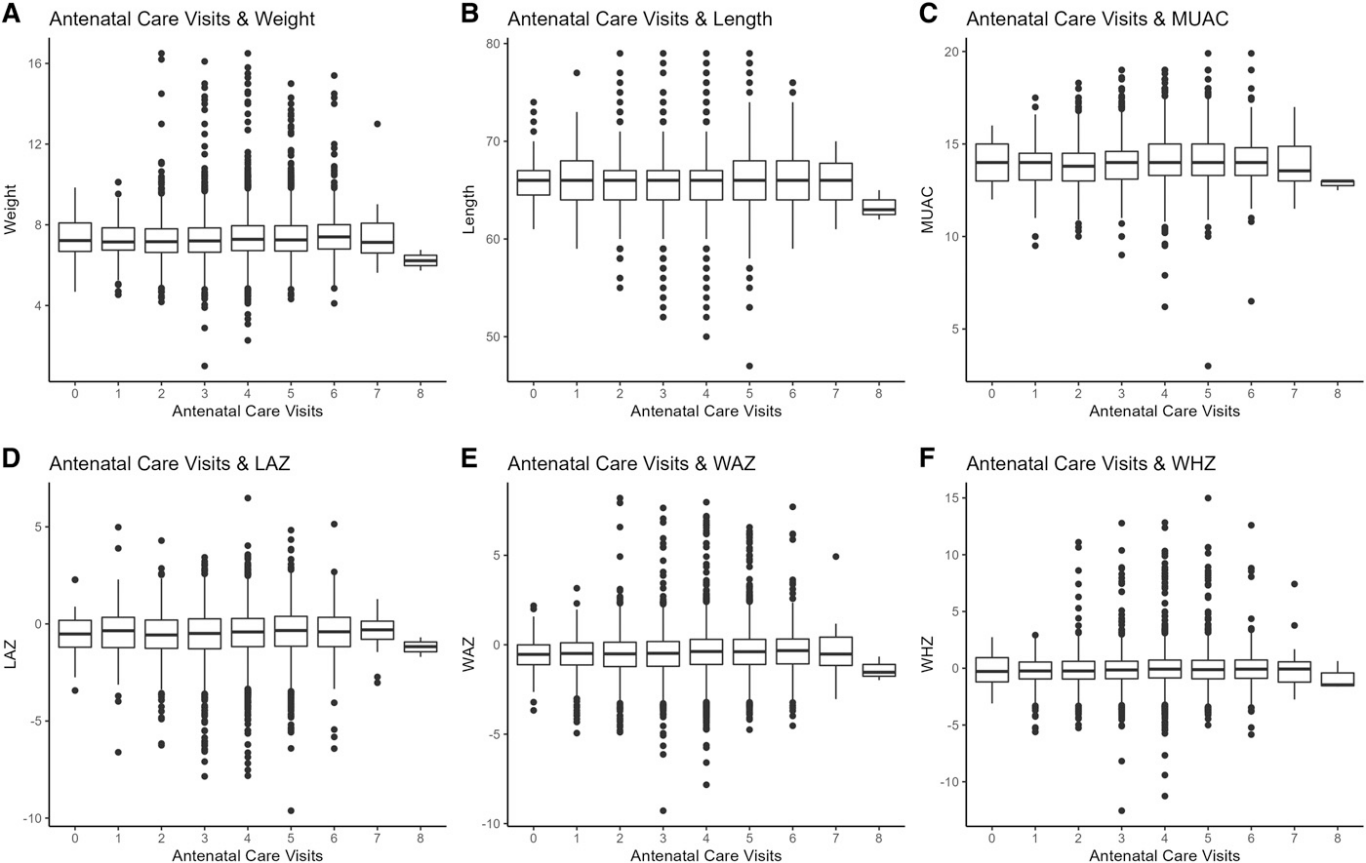
Distributions of growth measurements (weight, length, mid-upper-arm circumference [MUAC], length-for-age Z-score [LAZ], weight-for-age Z-score [WAZ], and weight-for-height Z-score [WHZ]) across different levels of antenatal care visits.

Anthropometric data at 6 months was not available for all infants, which resulted in 19,108 infants included in the analyses for most growth endpoints and 18,513 for the MUAC analyses. Figure 1 presents box plots of each growth endpoint by the number of ANC visits attended. Growth endpoints varied only slightly by number of ANC visits. In models adjusted for potential confounding variables, there was a 0.03-kg increase in infants’ mean weight (mean difference 0.03 kg, 95% CI 0.02–0.04), a 0.07-cm increase in infants’ mean length (mean difference 0.07 cm, 95% CI 0.02–0.12), a 0.04-cm increase in infants’ mean MUAC (mean difference 0.04 cm, 95% CI 0.01–0.07), a 0.04 SD increase in infants’ mean LAZ (mean difference 0.04 SD, 95% CI 0.02–0.07), a 0.04 SD increase in infants’ mean WAZ (mean difference 0.04 SD, 95% CI 0.02–0.05), and a 0.02 SD increase in infants’ mean weight-for-height Z-score (WHZ; mean difference 0.02 SD, 95% CI 0.00–0.04) for each additional ANC visit attended by the mother (Table 2). These mean differences in growth outcomes were statistically significant, except for WHZ.

**Table 2:** Unadjusted and adjusted mean differences of 6-month growth outcomes comparing mothers with varying antenatal care attendance

Six-Month Outcomes	*N* *	Unadjusted Mean Differences (95% CI)	*P*-Value	Adjusted Mean Differences (95% CI)	*P*-Value
Weight, kg	19,108	0.04 (0.03–0.06)	<0.001	0.03 (0.02–0.04)	<0.001
Length, cm	19,108	0.08 (0.04–0.11)	<0.001	0.07 (0.02–0.12)	0.008
MUAC, cm	18,513	0.06 (0.04–0.07)	<0.001	0.04 (0.01–0.07)	0.006
LAZ	19,108	0.04 (0.03–0.06)	<0.001	0.04 (0.02–0.07)	0.001
WAZ	19,108	0.05 (0.04–0.07)	<0.001	0.04 (0.02–0.05)	<0.001
WHZ	19,108	0.04 (0.02–0.05)	<0.001	0.02 (0.00–0.04)	0.118

LAZ = length-for-age Z-score; MUAC = mid-upper-arm circumference; WAZ = weight-for-age Z-score; WHZ = weight-for-height Z-score.

Adjusted for infant’s sex, maternal age, maternal education, urbanicity, region, season, pregnancy type, if the infant was breastfed, and if there was an onsite physician.

* *N*s in analysis vary slightly due to missingness.

In the sensitivity analyses where we categorized ANC visits into two categories (zero to three versus four or more), the estimates for the mortality and 6 months of age growth outcomes were similar to the original analyses (Tables 3 and 4). However, after adjusting for potential confounders, individuals whose mothers attended at least four or more ANC visits had 0.72 times the odds of infant mortality (adjusted OR 0.72, 95% CI 0.44, 1.18) compared with infants whose mothers attended less than 4 ANCs (Table 3). This finding was not statistically significant. Similar to the main analysis, mean differences in growth outcomes were statistically significant, except for WHZ (Table 4).

**Table 3:** Unadjusted and adjusted odds ratios (OR) of 6-month mortality comparing mothers with varying antenatal care (ANC) visit attendance

Variable	*N*	Univariate OR (95% CI)	*P*-Value	Adjusted OR (95% CI)	*P*-Value
No. of ANC visits (continuous variable)	20,835	0.87 (0.73–1.05)	0.15	0.89 (0.73–1.09)	0.27
No. of ANC visits (binary variable)*	20,835	0.68 (0.45–1.03)	0.07	0.72 (0.44–1.18)	0.20

Associations between ANC visits and mortality. Adjusted for infant’s sex, maternal age, maternal education, urbanicity, region, season, pregnancy type, if the infant was breastfed, and if there was an onsite physician.

* Reference: zero to three visits.

**Table 4:** Sensitivity analysis examining associations between antenatal care visits (zero to three and four or more) and 6-month growth outcomes.

Six-Month Outcomes*	*N* ^†^	Unadjusted Mean Differences (95% CI)	*P*-Value	Adjusted Mean Differences (95% CI)	*P*-Value
Weight, kg	19,108	0.11 (0.08–0.14)	<0.001	0.07 (0.03–0.10)	<0.001
Length, cm	19,108	0.18 (0.10–0.26)	<0.001	0.15 (0.02–0.27)	0.027
MUAC, cm	18,513	0.17 (0.14–0.21)	<0.001	0.13 (0.06–0.206)	0.001
LAZ	19,108	0.10 (0.06–0.14)	<0.001	0.10 (0.04–0.16)	0.002
WAZ	19,108	0.13 (0.10–0.16)	<0.001	0.09 (0.05–0.13)	<0.001
WHZ	19,108	0.09 (0.05–0.13)	<0.001	0.04 (0.01–0.09)	0.080

LAZ = length-for-age Z-score; MUAC = mid-upper-arm circumference; WAZ = weight-for-age Z-score; WHZ = weight-for-height Z-score.

Adjusted for infant’s sex, maternal age, maternal education, urbanicity, region, season, pregnancy type, if the infant was breastfed, and if there was an onsite physician.

* Reference: zero to three visits.

^†^ *N*s in analysis vary slightly because of missingness.

## DISCUSSION

This study aimed to examine the association between ANC attendance and infant mortality and growth outcomes at 6 months of age using data from the NAITRE trial conducted in Burkina Faso.^12^ After adjusting for potential confounders, we found no evidence that increased number of ANC visits were associated with infant mortality. There was evidence that increased ANC attendance was associated with better growth outcomes at 6 months compared with infants whose mothers had lower ANC attendance; however, differences were small and may not be clinically meaningful. Given the large sample size of the trial, this analysis was powered to detect very small differences in continuous endpoints. Any effect of ANC on infant growth outcomes is likely because higher ANC attendance results in improved birth outcomes, leading to better growth trajectories.^15^ However, given the very small absolute differences in growth endpoints, longer-term effects of ANC attendance on infants may be minimal. This suggests that ANC attendance alone may not be sufficient to significantly influence infant mortality in this setting and may not have large effects on infant growth outcomes up to 6 months of age. Other factors, such as the distance to a health clinic, may also play a role in reducing mortality and improving growth outcomes.^16^

Studies commonly evaluate birth weight when examining the role of ANC attendance on weight.^17^ This includes the previous NAITRE analysis examining the association of ANC attendance and birthweight.^5^ Even though these studies found that higher ANC attendance is associated with a lower risk of LBW among infants, these studies typically do not evaluate longer-term outcomes.^5^^,^^17^ The results of the current study extend the literature of the effect of ANC on infant outcomes by evaluating longer-term endpoints. Although there may be modest benefits of ANC on infant outcomes after birth, differences observed in this analysis were small. Future studies should examine the associations of ANC attendance on other health outcomes beyond birth weight and infant mortality, especially health outcomes occurring later in a child’s life.

This study has several limitations. First, the small number of infant deaths in the study limits the power of the analyses examining the associations between ANC attendance and infant mortality. Second, the large study sample size provides statistical power to detect very small differences in growth outcomes. Most differences in growth endpoints were small. Small differences in outcomes may not have a meaningful influence on actual health outcomes or may not be clinically relevant. In addition, small differences may be more likely to be attributable to bias, including unmeasured confounding. Third, the study enrollment criterion requiring infants to weight at least 2,500 g by 27 days of age may have resulted in selection bias where the most vulnerable infants were likely excluded. If infants born to mothers with fewer ANC visits more often died before day 27 or weighed less than 2,500 g, this could have resulted in differential selection into the study. Healthier infants (those who survive and weigh more) from mothers with fewer ANC visits would be represented in higher proportions, which would bias the results toward null. This is important to note because mothers of premature infants would have consequently had shorter pregnancies and therefore fewer ANC visits. Fourth, due to limitations of resources in the facilities in which infants were enrolled in this trial, we were unable to measure gestational age and thus are unable to comment on whether low birthweight infants in this analysis were premature or full term but small for gestational age, such as infants with intrauterine growth restriction.

This study was an analysis of a randomized controlled trial, and thus adjustments for confounding were limited to variables that were available in the dataset. There may have been several unmeasured confounders that were not accounted for in analyses, including distance from the family’s residence to the healthcare facility and measures of SES beyond maternal education. Although we tried to account for proxies of wealth by adjusting for urbanicity and maternal education, residual confounding by SES could lead to biased results as wealthier individuals are likely to have fewer barriers to accessing healthcare and may have access to better-equipped facilities. The study also did not measure smoking during pregnancy, which could be a potential confounding factor. Smoking is known to have adverse effects on pregnancy outcomes and infant health, and its exclusion as a variable may introduce bias into the results.

Measurement error could have influenced the validity of the results. Although the nurses who collected the anthropometric measurements and other health data were trained, the equipment was standardized, and we monitored data in real time, there is the potential for human error or equipment malfunction during data collection. However, it is unlikely any potential measurement error had major effects on the results because it is most likely very small and unlikely to be differential by exposure category. Anthropometric data were missing for some infants whose 6-month visit was conducted via phone call rather than an in-person visit in the clinic. If missing anthropometric data was differential by number of ANC visits, this could introduce additional bias to the results. Only three women in the study attended eight ANC visits. As a result, this study cannot directly comment on outcomes among women following the WHO’s recommendations of at least eight ANC contacts. Lastly, the findings of this study may have limited generalizability to other settings. The study was conducted in a low-income country among a specific population in Burkina Faso, which may have unique characteristics that differ from other regions or high-income countries. Therefore, caution should be exercised when extrapolating these findings to other populations or settings.

## CONCLUSION

In conclusion, this study found that higher ANC attendance was associated with improved growth outcomes at 6 months of age, although the absolute differences were small. These findings emphasize the importance of ANC in promoting healthy growth and development during the early stages of life. It is noteworthy that although the WHO recommends women attend at least eight ANC visits, only three of the mothers in this study reached this goal. Future research and policy changes should focus on identifying the specific interventions within ANC that are most effective in improving infant health outcomes and explore strategies to enhance the quality and coverage of ANC services in resource-limited settings.

## Supplemental Materials

10.4269/ajtmh.23-0659 Supplemental Materials
