# Supplementary material for: Associations between Antenatal Care Visit Attendance and Infant Mortality and Growth
Source: Am J Trop Med Hyg. 2024 Apr 16;110(6):1270–5. doi: 10.4269/ajtmh.23-0659 (PMC11154033; doi:10.4269/ajtmh.23-0659)
Supplement: Supplemental Materials [file tpmd230659.SD1.pdf]

Supplemental Figure & Table

Figure 1: Mean growth by antenatal care visit.

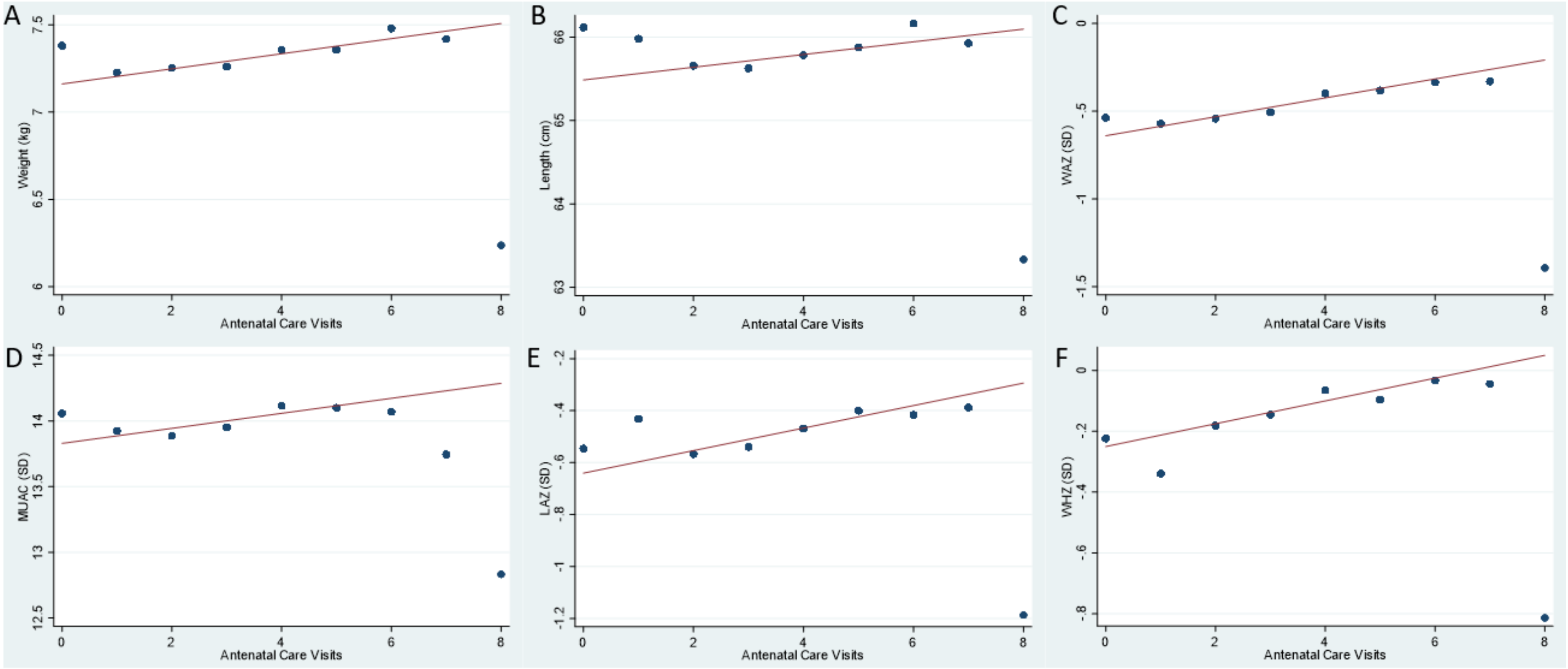

A = Weight; B = Length; C = WAZ; D = MUAC; E = LAZ; F = WHZ.

Table 1. Sensitivity analysis examining associations between antenatal care visits and mortality at 6 months among the control group only.

	**Univariate Odds Ratio (95% CI)**	**P-value**	**Multivariable Adjusted Odds Ratio (95% CI)**	**P-value**
No. of antenatal care visits	0.96 (0.74, 1.24)	0.740	0.99 (0.77, 1.28)	0.956

Adjusted for child's sex, maternal age, maternal education, urbanicity, region, season, pregnancy type, if the infant was breastfed, and if there was an onsite physician.

N = 10,435.
